# Complicated infective endocarditis: a case series

**DOI:** 10.1186/s13256-017-1274-7

**Published:** 2017-05-08

**Authors:** Joo Seop Kim, Min-Kyung Kang, A. Jin Cho, Yu Bin Seo, Kun Il Kim

**Affiliations:** 1 Division of Cardiology, Kangnam Sacred Heart Hospital, Hallym University Medical Center, Seoul, South Korea; 2 Division of Nephrology, Kangnam Sacred Heart Hospital, Hallym University Medical Center, Seoul, South Korea; 3 Division of Infection, Kangnam Sacred Heart Hospital, Hallym University Medical Center, Seoul, South Korea; 4 Division of Cardiothoracic Surgery, Kangnam Sacred Heart Hospital, Hallym University Medical Center, Seoul, South Korea

**Keywords:** Complications, Infective endocarditis, Mycotic aneurysm

## Abstract

**Background:**

Infective endocarditis is associated with not only cardiac complications but also neurologic, renal, musculoskeletal, and systemic complications related to the infection, such as embolization, metastatic infection, and mycotic aneurysm.

**Case presentation:**

We report three cases (the first patient is Chinese and the other two are Koreans) of complicated infective endocarditis; two of the cases were associated with a mycotic aneurysm, and one case was associated with a splenic abscess. One case of a patient with prosthetic valve endocarditis was complicated by intracerebral hemorrhage caused by mycotic aneurysm rupture. A second case of a patient with right-sided valve endocarditis associated with a central catheter was complicated by an abdominal aortic mycotic aneurysm. The third patient had a splenic infarction and abscess associated with infected cardiac thrombi.

**Conclusions:**

Complicated infective endocarditis is rare and is associated with cardiac, neurologic, renal, musculoskeletal, and systemic complications related to infection, such as embolization, metastatic infection, and mycotic aneurysm. Infective endocarditis caused by *Staphylococcus aureus* is more frequently associated with complications. Because the mortality rate increases when complications develop, aggressive antibiotic therapy and surgery, combined with specific treatments for the complications, are necessary.

**Electronic supplementary material:**

The online version of this article (doi:10.1186/s13256-017-1274-7) contains supplementary material, which is available to authorized users.

## Background

Infective endocarditis (IE) is associated with not only cardiac complications but also neurologic, renal, musculoskeletal, and systemic complications related to infection, such as embolization, metastatic infection, and mycotic aneurysm (MA) [1]. We report three cases of patients with complicated IE; two were associated with MA, and one was associated with splenic abscess.

## Case presentations

### Patient 1

**Fig. 1:**
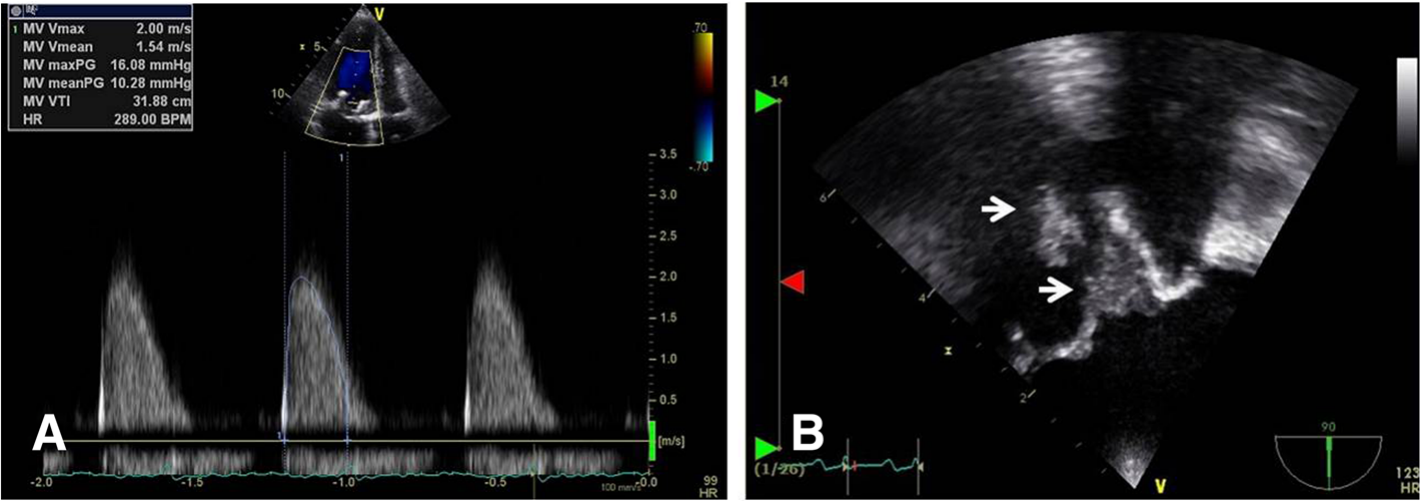
Transthoracic echocardiograms showing an elevated mean diastolic pressure gradient (**a**) of 10.3 mmHg and a hyperdynamic echogenic mass attached to the prosthetic MV (**b**) (*white arrows*)

**Fig. 2:**
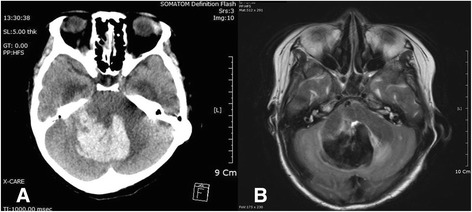
Brain computed tomography (**a**) and magnetic resonance imaging (**b**) revealing acute hemorrhage with perilesional edema in the bilateral cerebellum causing obstructive hydrocephalus, suggestive of mycotic aneurysmal rupture



A 57-year-old Chinese woman presented to our hospital with generalized weakness. Her medical history included hypertension and early liver cirrhosis caused by chronic viral hepatitis C (platelet count 104,000/μl). She had undergone mitral valve replacement (MVR) with a Hancock II 27-mm prosthesis (Medtronic, Minneapolis, MN, USA) for mitral valve prolapse 1 month earlier. Her blood pressure was 100/60 mmHg, her breathing rate was 12 breaths/minute, her heart rate was 121 beats/minute, and her body temperature was 36.5 °C. She appeared acutely ill and was dehydrated. The result of an initial chest x-ray was normal, and the patient’s electrocardiogram showed sinus tachycardia. Transthoracic echocardiography (TTE) showed normal prosthetic valve motion without evidence of vegetation or paravalvular leakage, but the patient’s mean diastolic pressure gradient was elevated at 10.3 mmHg (Fig. 1a) [2]. She had been discharged on warfarin and other medications after successful MVR without complications. Transesophageal echocardiography (TEE) (Fig. 1b and Additional file 1: Video 1) showed hyperdynamic echogenic material attached to the prosthetic MV. During preparation for redo surgery with administration of appropriate antibiotics, the patient suddenly had a generalized seizure with decreased mentation. Brain computed tomography (CT) and magnetic resonance imaging (Fig. 2a, b) revealed acute hemorrhage with perilesional edema in the bilateral cerebellum causing obstructive hydrocephalus, suggestive of MA rupture. She was transferred to the intensive care unit with an indwelling external ventricular drain (EVD) for monitoring. We performed a redo MVR with a Hancock II 27-mm prosthesis after resolution of her hemorrhage.

**Additional file 1:** TEE showed a hyper-dynamic echogenic mass attached to the prosthetic mitral valve. (WMV 804 kb)


### Patient 2

**Fig. 3:**
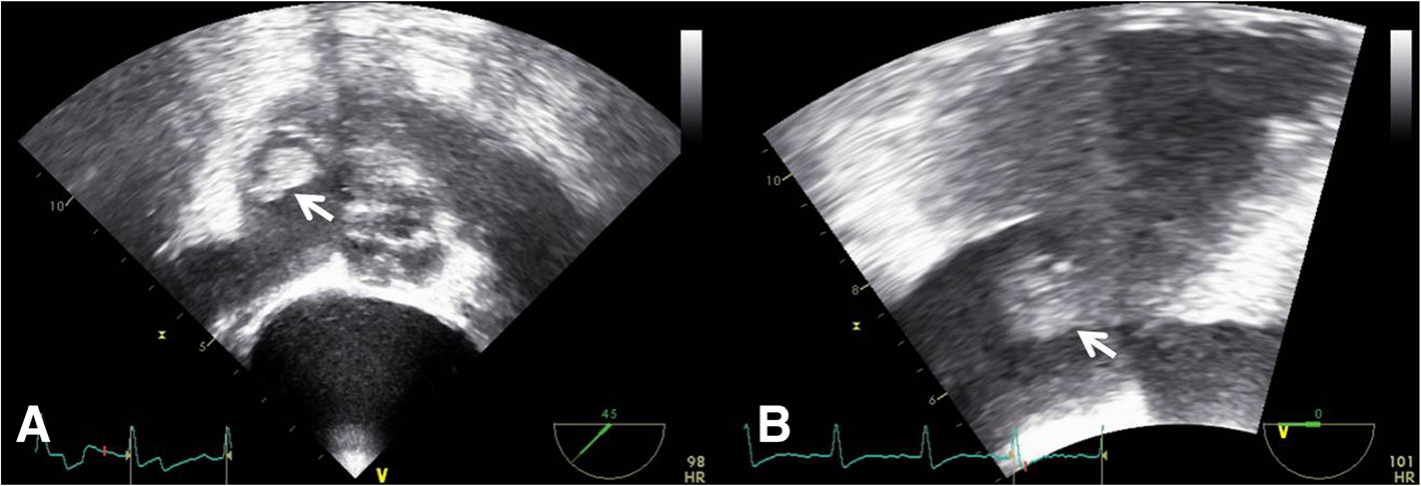
**a** and **b** Transesophageal echocardiography revealing a globular, mobile, echogenic mass (1.91 × 1.0 cm; *arrows*) attached to the tricuspid valve

**Fig. 4:**
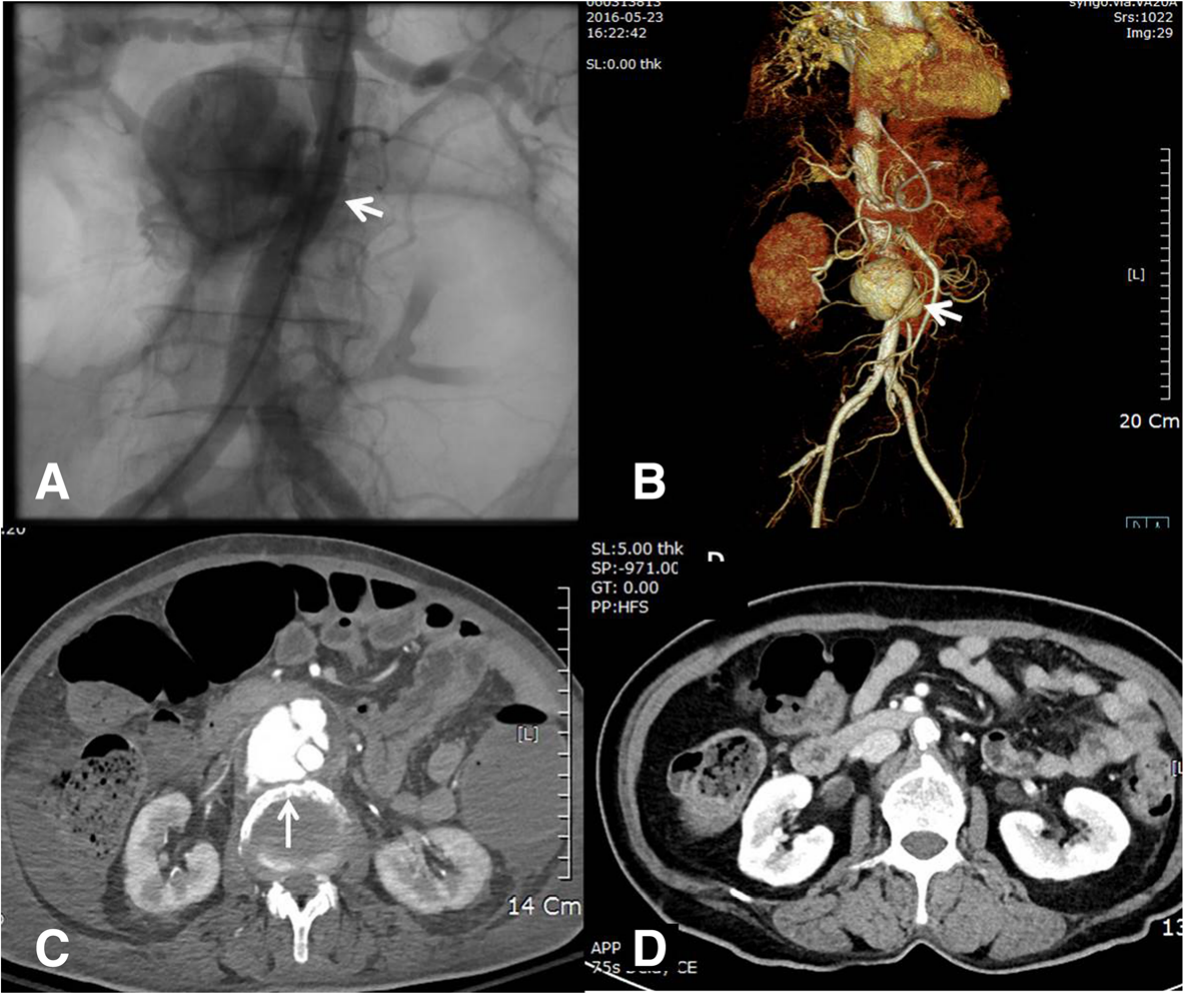
A large saccular aneurysm was detected in the infrarenal abdominal aorta by aortography (**a**, *white arrow*) and computed tomography (**b** and **c**, *white arrows*). The aneurysm had not been present 2 years previously (**d**)



A 70-year-old Korean woman presented with generalized weakness and headache. Her medical history included diabetes mellitus and hypertension. Her physical examination revealed her blood pressure was 163/81 mmHg and her pulse rate was 91 beats/minute. Brain CT revealed a chronic left subdural frontotemporal hemorrhage. After burr hole trephination was performed, generalized edema developed because of acute kidney injury (creatinine level 0.86 → 1.89 mg/dl). A peripherally inserted central catheter (PICC) was placed. The patient developed a fever (38.1 °C) after 3 weeks, without a definite source of infection. TEE revealed a globular, mobile, echogenic mass (1.91 × 1.0 cm) (Fig. 3a, b) attached to the tricuspid valve. Blood cultures revealed *Staphylococcus aureus* sensitive to vancomycin. The patient’s fever subsided after treatment with antibiotics, but a vegetation and persistent septicemia were noted after 2 weeks. We performed coronary angiography prior to possible valve surgery and observed no significant coronary obstruction, but a large saccular aneurysm was detected in the infrarenal abdominal aorta (Fig. 4a and Additional file 2: Video 2). CT indicated this was a newly developed abdominal aortic aneurysm (maximum diameter 5.2 cm) (Fig. 4b, c) that had not been present 2 years previously (Fig. 4d). The appearance was suggestive of an MA associated with IE. We recommended valve surgery and endovascular stenting or surgical removal of the MA in sequence, but the patient’s guardians refused. The patient was discharged to a private convalescent hospital and was lost to follow-up. We suspect IE developed in association with PICC placement and that persistent septicemia, despite use of proper antibiotics, led to an MA.

**Additional file 2:** A large saccular aneurysm was detected in the infrarenal abdominal aorta by aortography. (WMV 2144 kb)


### Patient 3

**Fig. 5:**
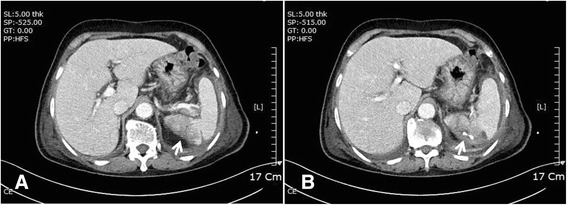
Computed tomography shows splenic infarction (**a**, *white arrow*) and abscess with percutaneous drainage (**b**, *white arrow*)

**Fig. 6:**
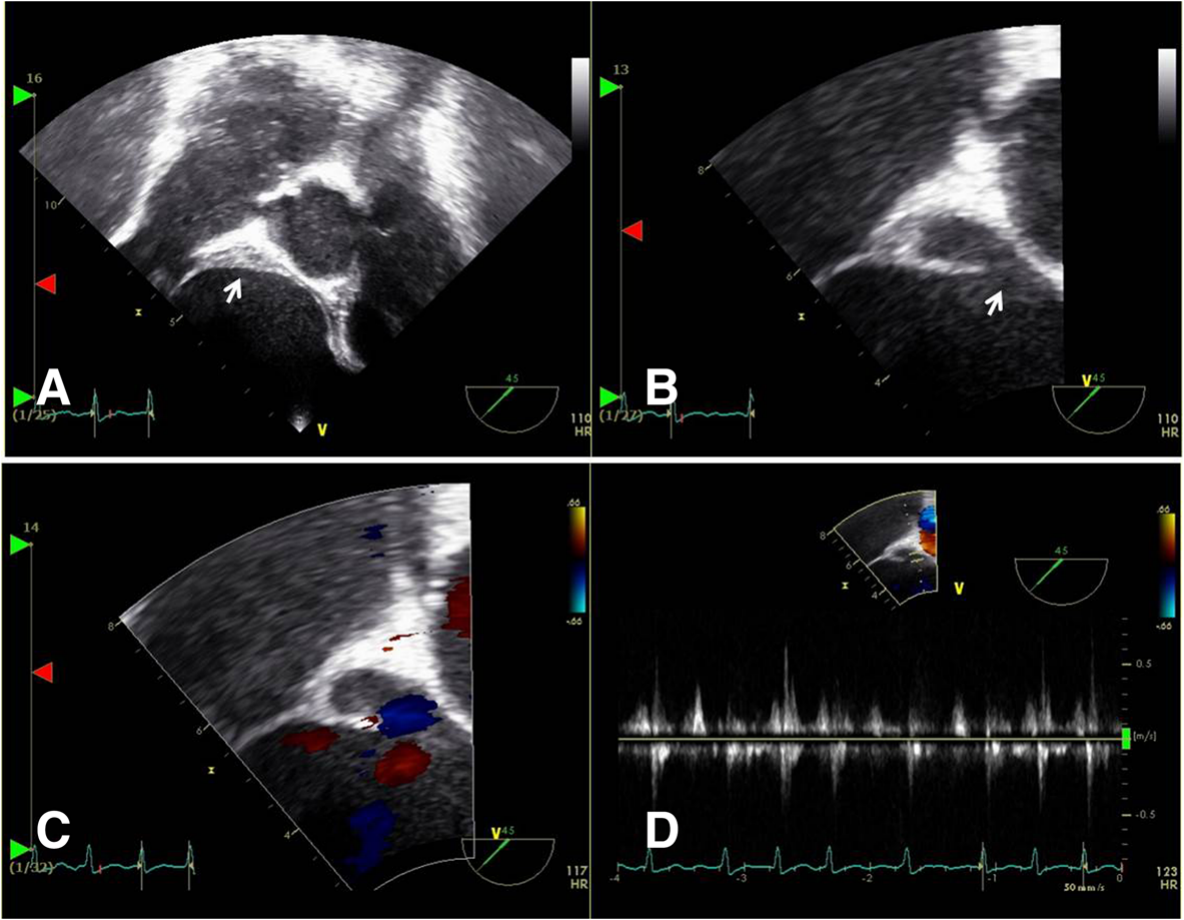
Transesophageal echocardiography shows a thickened nonhomogeneous area with echo-dense appearance around the aortic root (**a**, *white arrow*), as well as discontinuous endocardial tissue (**b**, *white arrow*) with flow communication detected by color and pulsed wave Doppler ultrasound (**c** and **d**)

**Fig. 7:**
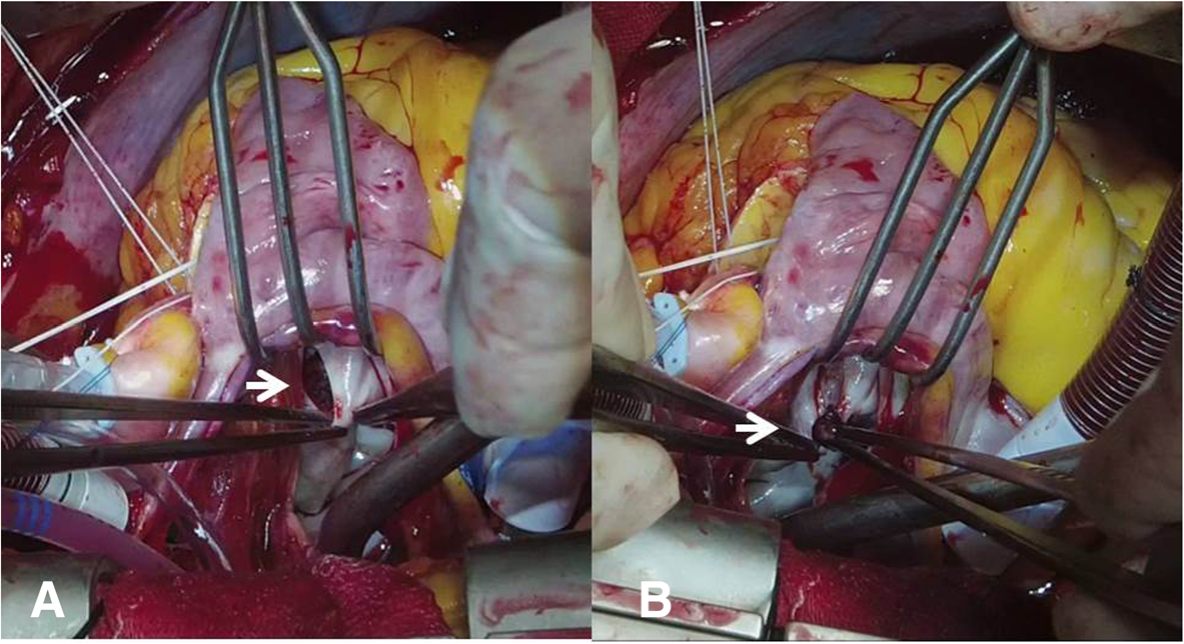
Intraoperative views show a weblike structure with interruption of endocardial tissue continuity (**a**, *white arrow*) and that thrombi were present in the pocket (**b**, *white arrow*)



A 76-year-old Korean woman with acute onset of flank pain caused by splenic infarction and abscess was transferred to our hospital with percutaneous drainage (Fig. 5). She had been diagnosed with a stroke in our hospital 3 months earlier. Other than fever (38.1 °C), she had stable vital signs. Although splenic infarction or embolism is common, splenic abscess is rare. In every patient diagnosed with splenic infarction, a search for the possible source of emboli should be performed, and IE is the most common cause [3]. There was no evidence of IE in this patient, but slightly increased mitral regurgitation (grade 1–2) (Additional file 3: Video 3 and Additional file 4: Video 4) was noted by TTE. TEE revealed a thickened, nonhomogeneous area with an echo-dense appearance around the aortic root (Fig. 6a) and discontinuous endocardial tissue (Fig. 6b) with flow communication detected by color and pulsed wave Doppler ultrasound (Fig. 6c, d). This patient needed surgery for locally uncontrolled infection [4]. A weblike structure with interruption of endocardial tissue continuity was noted (Fig. 7a), and thrombi were observed within the pocket (Fig. 7b). The patient recovered fully and was discharged.

**Additional file 3:** TTE showed mild mitral regurgitation. (WMV 1254 kb)




**Additional file 4:** TTE showed increased mitral regurgitation. (WMV 1692 kb)


## Discussion

IE is associated with cardiac, neurologic, renal, and musculoskeletal complications. Predisposing factors include the infecting pathogen, duration of illness prior to therapy, and underlying comorbidities [1]. IE caused by *S. aureus* is associated with complications more frequently than other pathogens [5].

MA can develop in the cerebral or systemic circulation in the setting of IE [6], and cerebral hemorrhage caused by stroke or a ruptured MA can cause neurologic complications. Direct bacterial inoculation, bacteremic seeding, contiguous infection, and septic emboli are the sources of MA. Intracranial MA usually involves more distal portions of the middle cerebral artery, as in patient 1, and unruptured aneurysms may be managed with antibiotics alone. However, ruptured aneurysms should be managed with a combination of antibiotics and surgery [6]. Patient 1 developed intracranial hemorrhage and a seizure; we initially attempted conservative treatment with EVD and close monitoring in the intensive care unit, and then we performed a redo MVR after stabilization [4]. The perioperative mortality rate for infected aortic aneurysms is 15% to 20% [7]. Survival is better for an infrarenal abdominal aortic aneurysms than for noninfrarenal aneurysms (96% versus 57%, respectively) [8]. Therefore, we suggest debridement of an infected infrarenal aortic aneurysm, along with extraanatomic reconstruction. The guardians of patient 2 refused all surgery because of the high morbidity and mortality risk.

In contrast to relatively common splenic infarctions in patients with embolic events, splenic abscesses are very rare and fatal complications of IE [3]. The treatments of choice are antibiotics, splenectomy, and valve replacement surgery. After patient 3 was stabilized with drainage of a splenic abscess, we decided to perform valve replacement surgery and debridement of the perforated intracardiac abscess pocket. The aortic and mitral valves were relatively clean, except for degenerative changes, but infected thrombi were noted in a phlegmon.

## Conclusions

IE is associated with cardiac, neurologic, renal, musculoskeletal, and systemic complications related to the infection (embolization, metastatic infection, and MA). Predisposing factors include the infecting pathogen, duration of illness, prior therapy, and underlying comorbidities. Complications can occur before, during, and after completion of therapy. IE caused by *S. aureus* is associated with complications more frequently. Because the mortality rate increases with complications, aggressive antibiotic therapy combined with surgery and other specific treatments for complications is necessary.

## Abbreviations

CT: Computed tomography
EVD: External ventricular drain
IE: Infective endocarditis
MA: Mycotic aneurysm
MVR: Mitral valve replacement
PICC: Peripherally inserted central catheter
TEE: Transesophageal echocardiography
TTE: Transthoracic echocardiography
